# Mutational Signatures in Cancer (MuSiCa): a web application to implement mutational signatures analysis in cancer samples

**DOI:** 10.1186/s12859-018-2234-y

**Published:** 2018-06-14

**Authors:** Marcos Díaz-Gay, Maria Vila-Casadesús, Sebastià Franch-Expósito, Eva Hernández-Illán, Juan José Lozano, Sergi Castellví-Bel

**Affiliations:** 1 Gastroenterology Department, Hospital Clínic, Institut d’Investigacions Biomèdiques August Pi i Sunyer (IDIBAPS), Centro de Investigación Biomédica en Red de Enfermedades Hepáticas y Digestivas (CIBEREHD, University of Barcelona, Barcelona, Spain; 2 Bioinformatics Platform, Centro de Investigación Biomédica en Red de Enfermedades Hepáticas y Digestivas (CIBEREHD), Barcelona, Spain; 3 Present Address: Gene Regulation, Stem Cells and Cancer Program, Centre for Genomic Regulation (CRG), The Barcelona Institute of Science and Technology (BIST), Barcelona, Spain

**Keywords:** Mutational signatures, COSMIC database, Single nucleotide variants, Cancer genomics, Web tool, Shiny, R language

## Abstract

**Background:**

Mutational signatures have been proved as a valuable pattern in somatic genomics, mainly regarding cancer, with a potential application as a biomarker in clinical practice. Up to now, several bioinformatic packages to address this topic have been developed in different languages/platforms. MutationalPatterns has arisen as the most efficient tool for the comparison with the signatures currently reported in the Catalogue of Somatic Mutations in Cancer (COSMIC) database. However, the analysis of mutational signatures is nowadays restricted to a small community of bioinformatic experts.

**Results:**

In this work we present Mutational Signatures in Cancer (MuSiCa), a new web tool based on MutationalPatterns and built using the Shiny framework in R language. By means of a simple interface suited to non-specialized researchers, it provides a comprehensive analysis of the somatic mutational status of the supplied cancer samples. It permits characterizing the profile and burden of mutations, as well as quantifying COSMIC-reported mutational signatures. It also allows classifying samples according to the above signature contributions.

**Conclusions:**

MuSiCa is a helpful web application to characterize mutational signatures in cancer samples. It is accessible online at http://bioinfo.ciberehd.org/GPtoCRC/en/tools.html and source code is freely available at https://github.com/marcos-diazg/musica.

**Electronic supplementary material:**

The online version of this article (10.1186/s12859-018-2234-y) contains supplementary material, which is available to authorized users.

## Background

Mutational processes in somatic cells are mainly led by endogenous or exogenous mutagenic agents, as well as errors in DNA replication or repair machineries. Any type of agent or defect is responsible for a specific footprint in the form of a different burden and pattern of mutations. Some of them are historically well-known, as in the case of ultraviolet light exposure and its association with C > T and CC > TT substitutions caused by pyrimidine dimers [1].

In recent years, a new methodology has arisen on this field. Mutational signatures framework enables the association of patterns of mutations with cellular processes and external agents causing them [2]. Since all cancers are caused by somatic mutations, this methodology has the potential to provide insight into their underlying biological processes and become a biomarker in clinical practice [3]. It is based on a computational implementation of non-negative matrix factorization (NMF) considering more than 10,000 cancer samples [4, 5]. Using the information of somatic single nucleotide variants (SNVs), a series of mutational profiles are extracted. These profiles take into account not only substituted nucleotides (all replacements are referred to by the pyrimidine of the mutated Watson-Crick base pair) but also the 5′ and 3′ adjacent bases. A total of 96 possibilities are evaluated, allowing to detect processes responsible for the same substitutions but in different contexts. According to the current information of the Catalogue of Somatic Mutations in Cancer (COSMIC) database [6], thirty mutational signatures have already been identified across 40 different types of human cancer. This methodology has the potential to reconstruct the mutational spectrum of any cancer sample with sufficient accuracy. This reconstruction is based on the combination of the different signatures contributions. Thus, it constitutes the imprint on the genome of specific mutagenic agents or genetic defects, each represented by a specific signature.

Several bioinformatic approaches have been developed to address mutational signature analysis using different platforms and programming languages. Including some commonalities such as the 96-mutation profile plotting (6 different nucleotide substitutions * 16 different 3-mer contexts), different packages have been recently developed for de novo signature extraction and contribution of known signatures. This is extremely important regarding the possibility of using this methodology in clinical practice. In this context, it would be convenient to perform the analysis at sample resolution, and this is only achievable by comparison with a set of established signatures. MutationalPatterns is an R/Bioconductor package that covers the whole spectrum of functionalities required for mutational signatures framework implementation [7]. It allows the extraction of de novo signatures using the original NMF algorithm, like former R packages pmsignature [8] and Somatic Signatures [9], and Galaxy tool MutSpec [10]. In addition, it also permits the quantification of COSMIC-reported signatures by finding their optimal linear combination. This process is performed approximately 400 times faster than deconstructSigs [11], the only package also covering this functionality [7]. MutationalPatterns has been proved useful in recent studies, both in the identification of somatic mutational profiles [12] and in the characterization of known mutational signatures in human stem cells [13].

However, analysis of somatic mutational signatures remains currently inaccessible for a substantial proportion of the scientific community. Developed software is only useful for bioinformatic experts that should adapt it to their somatic analysis pipelines. Computational resources are also a big challenge, especially when the number of samples to handle is considerably high. In this regard, we have developed a web application to overcome these challenges. Mutational Signatures in Cancer (MuSiCa) allows an easy and quick analysis of mutational signatures in cancer samples, based on a user-friendly web environment adapted to the whole research community. It is mainly built on top of the MutationalPatterns package, so benefiting from its functionalities but also adding a graphical interface designed for non-specialized researchers. MuSiCa also presents some extra features specially designed for cancer samples characterization. Its main aim is to quantify known mutational signatures contribution at sample level, therefore facilitating the identification of the underlying mutational processes. The application also permits to perform an analysis for a complete cohort of cancer patients.

## Implementation

MuSiCa was developed using the Shiny framework, which enables the straightforward building of interactive web applications directly from R code [14]. It integrates different publicly available R packages in order to generate a convenient interface and computational efficiency to fluently handle somatic mutation data. Regarding mutational signature framework, MuSiCa uses the data available in COSMIC. Hitherto, the 30 signatures that have been validated and reported in this database have been considered, with the prospect of a future update which would also be transferred to the application. MuSiCa can be easily run online at http://bioinfo.ciberehd.org/GPtoCRC/en/tools.html. Source R code is freely available to download at https://github.com/marcos-diazg/musica, where the required dependencies to install the application are indicated.

**Fig. 1:**
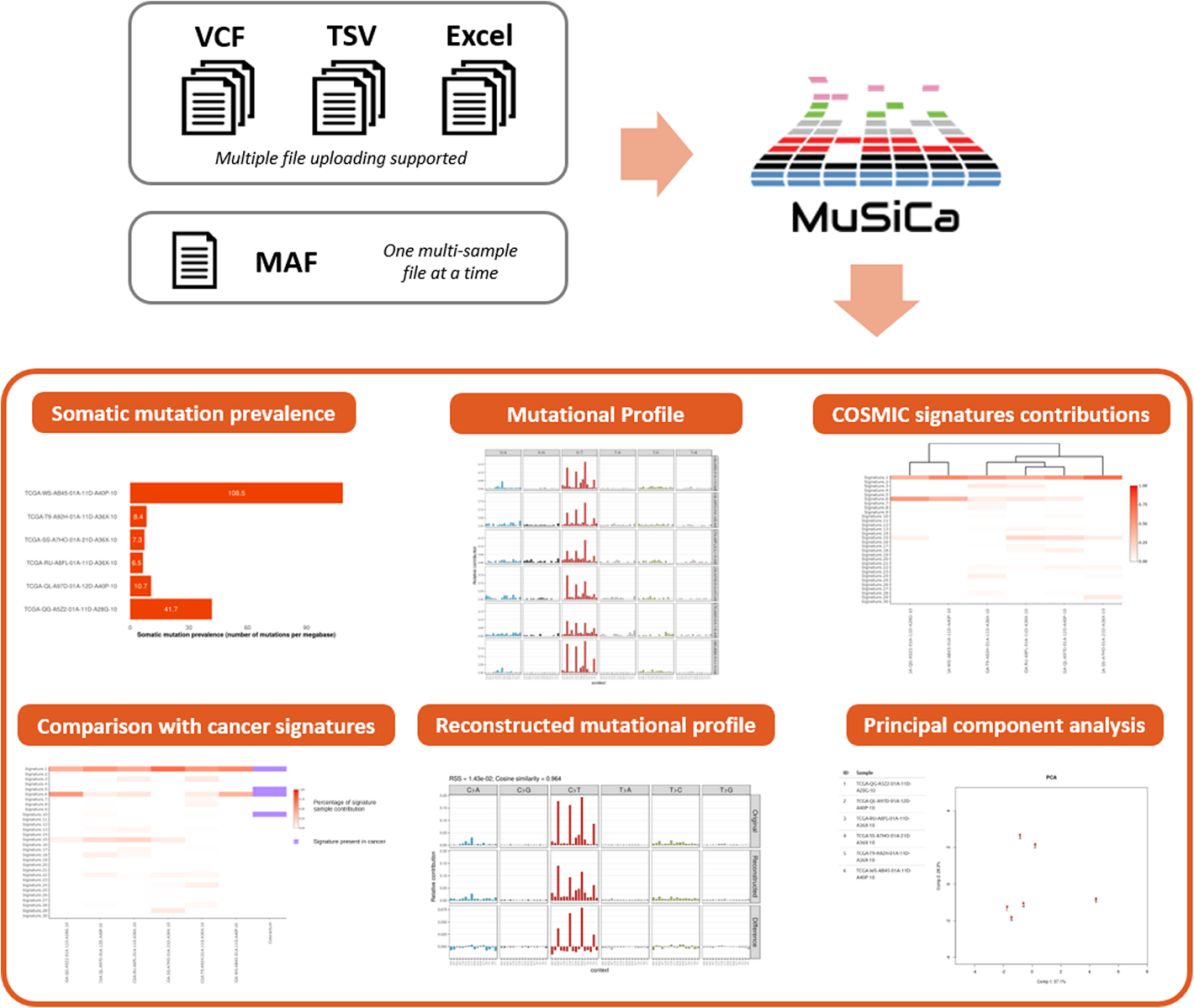
Analysis workflow of MuSiCa. Input options supported by the web application and output elements available as plots and data tables ready to download by the users

A typical workflow of MuSiCa application is presented in Fig. 1. It starts with the uploading of the files containing the somatic SNVs of the samples to analyze. Samples may be derived from international studies as ICGC/TCGA or directly provided by the users. The minimum information required is the chromosome and genomic position according to the human reference genome (UCSC GRCh38/hg38, GRCh37/hg19 and 1000genomes hs37d5 builds are supported), as well as the reference and alternative alleles for every mutation. Different file formats are permitted including the default for this kind of data, the Variant Call Format (VCF). Tab-Separated Values (TSV), Excel and Mutation Annotation Format (MAF) are also allowed. MAF format is commonly used for packing multi-sample data from the Genomics Data Commons projects. Multiple file uploading is allowed in the case of VCF, TSV and Excel formats, each containing the somatic mutations of one sample at a time. For MAF format, only one multi-sample file is allowed. A help modal is present in the MuSiCa website to clarify input format options to the users. The human reference genome build and the type of genomic study performed also need to be provided in order to correctly calculate the prevalence of somatic mutations (i.e. the number of mutations per megabase).

Output elements are displayed in six different tabs. They are presented in the form of publication-ready figures and tables that can be directly downloaded by the users in different formats. Firstly, mutation prevalence and profiling are presented for somatic mutation characterization. Regarding profiles, all possible SNVs considering the substituted base and the 5′ and 3′ adjacent nucleotides are depicted. Regarding the mutational signatures pattern, it is possible to visualize the contribution of COSMIC-reported signatures, as well as those associated with the distinct cancer types present in this database. The application also permits clustering samples and signatures according to the contributions using a distance measure based on Pearson correlation (1 – correlation value), as well as selecting which samples and cancer types are represented. A principal component analysis (PCA) plot is also presented when more than three samples are uploaded. Both clustering and PCA enable the classification of provided samples according to their quantification regarding known mutational signatures.

This process of signatures quantification is based on the least squares method. This method permits to find the optimal linear combination of the 30 signatures that minimize the residual sum of squares (RSS). Therefore, RSS is a measure of the efficiency of the original mutational profile reconstruction. MuSiCa presents an output tab where original and reconstructed profiles are depicted. RSS is also shown, as well as cosine similarity between both profiles. This value presents instead a direct measure of the correspondence between the two depicted profiles in a 0–1 range (identical profiles would have a value of 1). A value above 0.9 is considered as sufficient accuracy.

## Results and discussion

To assess the usability of the application, colon cancer SNV data from the NCI Genomic Data Commons was used. Four hundred thirty-three samples of this neoplasia were analyzed. They corresponded to the TCGA-COAD project. Somatic mutation data derived from TCGA projects was freely available in MAF format. As this is one of the supported input formats in MuSiCa, the application permitted to directly analyze this publicly available repository. Different upstream analysis workflows were available, using different somatic variant callers. MuTect2-derived data was selected in this example in accordance with GATK Best Practices [15].

Colorectal cancer is one of leading neoplasms worldwide considering mortality and morbidity. Regarding mutagenic agents, effects of environmental factors such as smoking are well-known. However, defects in key molecular pathways, especially those related with DNA repair, have been established as key factors in this neoplasm. Both malfunctioning of mismatch repair (MMR) genes and polymerases δ and ε are reported to affect colorectal carcinogenesis [16]. This is particularly important in the case of hypermutated tumors, defined as those having a mutation rate above 12 per 10^6^. This malfunctioning could be caused by somatic but also germline genetic alterations. Indeed, Lynch syndrome and Polymerase proofreading-associated polyposis are both hereditary colorectal cancer syndromes related to malfunctioning of previously indicated DNA repair pathways [17].

**Fig. 2:**
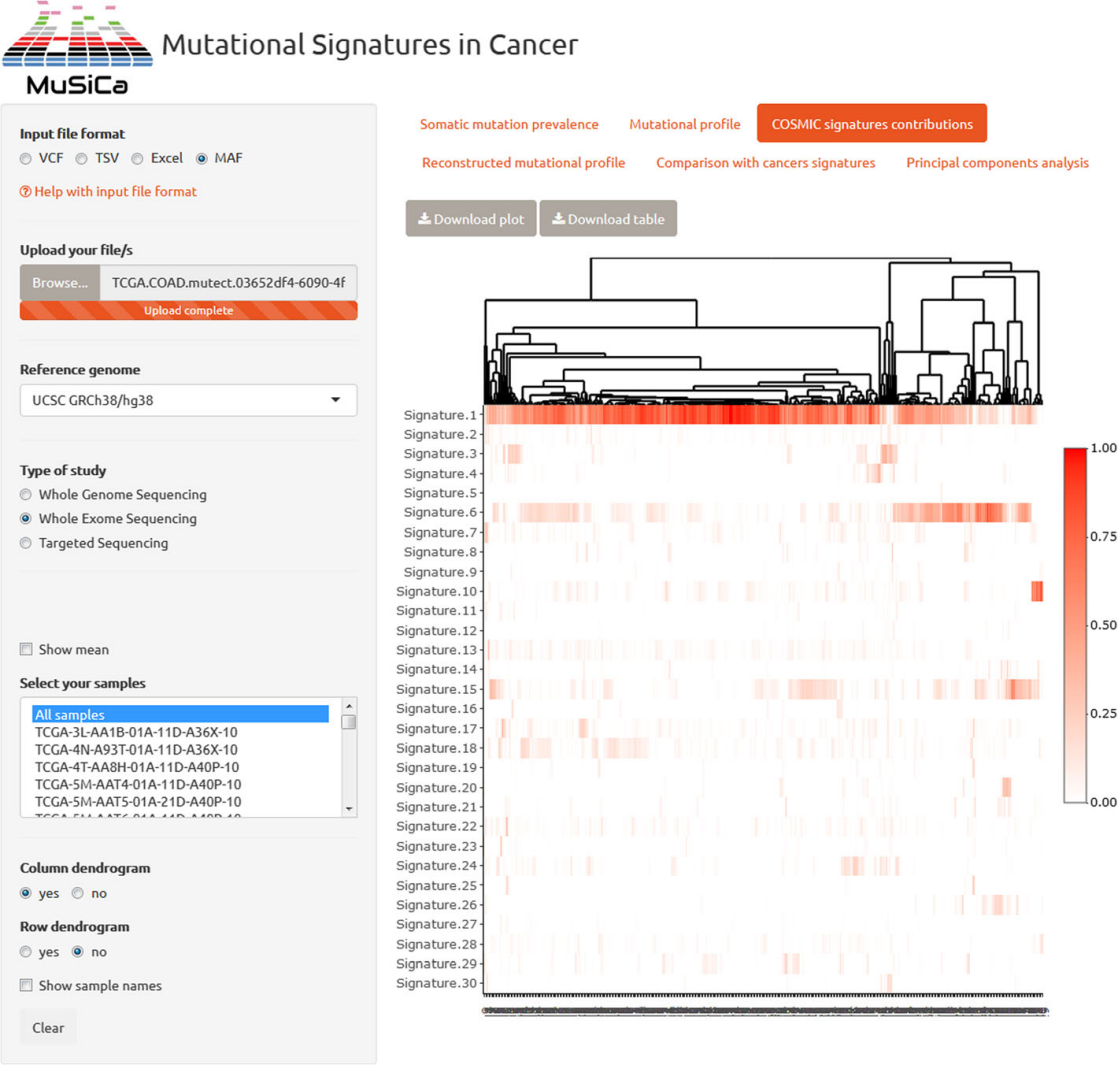
Capture of MuSiCa web application. Known signatures contribution (reported in COSMIC) of 433 TCGA-COAD colon cancer samples is shown. Input options are on the left-hand side and results appear on the main panel when the analysis is performed

Results of the analysis of colorectal cancer TCGA samples with MuSiCa are presented in Fig. 2 and Additional file 1. Regarding the quantification of COSMIC signatures, clustering discriminated at least three different subsets of colon cancer samples in this cohort. The group on the left, accounting for more than half of the samples, was mainly characterized by signature 1. This profile has been found in all cancer types and has been correlated with the age of cancer diagnosis. It is produced as an endogenous process derived from spontaneous deamination of 5-methylcytosine. The other two groups presented a higher level of signatures predominantly associated with MMR deficiency (signatures 6, 15 and 20) and defects in polymerase ε (signature 10). This is in agreement with microsatellite-unstable and *POLE*-mutated colon cancers [16]. However, they also showed the impact of age-associated signature 1. Therefore, this is a good example to realize how mutational signatures reconstruction highlighted the impact of the different underlying causes of mutations present in specific cancer samples. This fact could be a key evidence connecting to the carcinogenic process and even germline susceptibility to the neoplasm.

Regarding developed software for mutational signature analysis, some other tools were already available. In reference to bioinformatic packages, some different options were available as previously mentioned. MutationalPatterns has arisen as the most efficient tool enabling the comparison with the currently reported signatures. In recent years, some web applications have also been published in order to improve the accessibility to this methodology to the whole research community. Pmsignature was the first online application ready to apply mutational signatures framework [8]. However, it was intended just to extract new mutational signatures derived from the supplied samples, not allowing the comparison with known signatures. More recent examples include MutaGene, providing a huge computational framework regarding somatic cancer mutations [18]. It includes a large repository regarding mutational signatures, but it is more focused on the analysis of publicly available datasets than samples directly provided by the users. In fact, regarding this last point, it permits analyzing a set of samples but cannot generate analysis reports on a single sample level. mSignatureDB is a recent web implementation that allows for the first time to perform signature analysis on datasets directly uploaded by users [19]. Although it permits to quantify known mutational signatures contributions, it lacks some functionalities regarding sample classification, as clustering or PCA analysis. In addition, quantification process is based on deconstructSigs package, with the mentioned weakness on computational efficiency. To the best of our knowledge, no web application is able to characterize the burden of mutation of different cancer samples, as well as cluster and classify them according to their COSMIC-signatures quantification. Thus, MuSiCa becomes the most comprehensive tool available online for somatic characterization of cancer samples datasets directly provided by users.

## Conclusions

Our study shows the potential of the mutational signature framework as a biomarker in cancer and the simplicity and usefulness of our implementation. It is also remarkable that MuSiCa allows the analysis at sample level, which is mandatory regarding future clinical implementation of this methodology. Direct accessibility via web, user-friendly environment and computational performance are key factors of our application.

## Availability and requirements

- Project name: MuSiCa
- Project home page: https://github.com/marcos-diazg/musica
- Operating system(s): Platform-independent
- Programming language: R, Shiny
- Other requirements: Internet connectivity
- License: MIT License
- Any restrictions to use by non-academics: No

## Additional file

Additional file 1: **Figure S1**. Somatic mutational prevalence in MuSiCa web app. **Figure S2**. Mutational profile representation in MuSiCa web app. **Figure S3**. Reconstruction of mutational profile in MuSiCa web app. **Figure S4**. Comparison with cancer signatures in MuSiCa web app. **Figure S5**. Principal component analysis in MuSiCa web app. (PDF 3039 kb)

## Abbreviations

COSMIC: Catalogue of somatic mutations in cancer
MAF: Mutation annotation format
MMR: Mismatch repair
NMF: Non-negative matrix factorization
PCA: Principal component analysis
RSS: Residual sum of squares
SNV: Single nucleotide variant
TSV: Tab-separated values
VCF: Variant call format
