# Supplementary material for: Mutational Signatures in Cancer (MuSiCa): a web application to implement mutational signatures analysis in cancer samples
Source: BMC Bioinformatics. 2018 Jun 14;19:224. doi: 10.1186/s12859-018-2234-y (PMC6001047; doi:10.1186/s12859-018-2234-y)
Supplement: Supplementary file 1 — Figure S1. Somatic mutational prevalence in MuSiCa web app. Figure S2. Mutational profile representation in MuSiCa web app. Figure S3. Reconstruction of mutational profile in MuSiCa web app. Figure S4. Comparison with cancer signatures in MuSiCa web app. Figure S5. Principal component analysis in MuSiCa web app. (PDF 3039 kb) [file 12859_2018_2234_MOESM1_ESM.pdf]

Supplementary Material

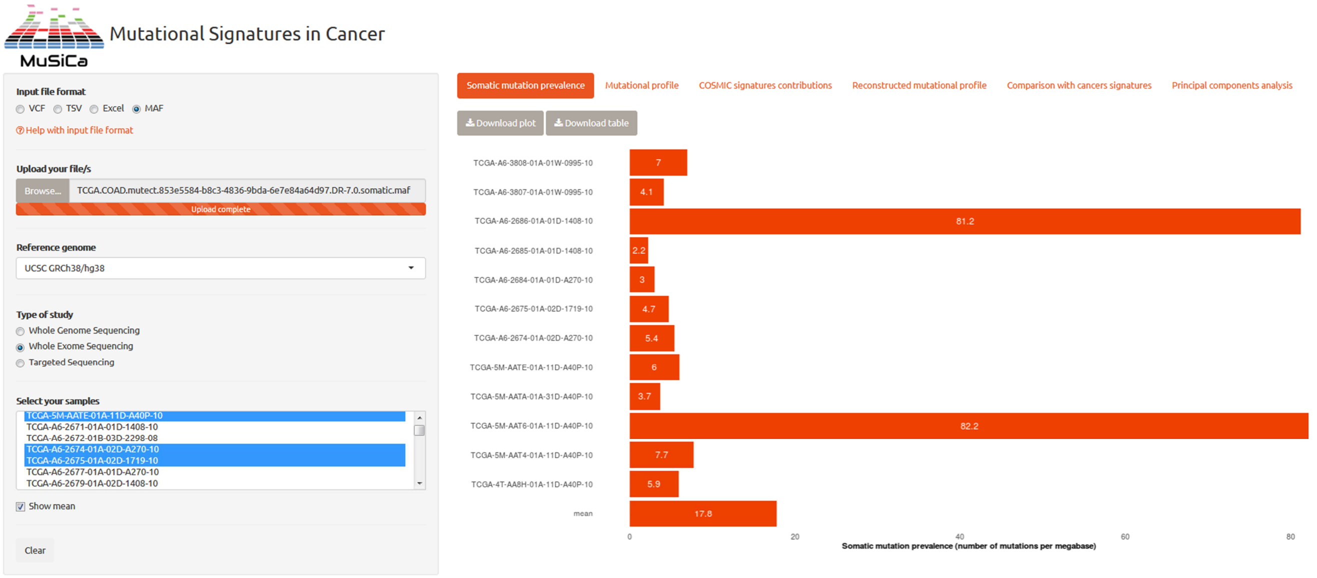

Supplementary Fig. 1. Somatic mutational prevalence in MuSiCa web app. Mutational burden output tab showing a subset of the TCGA-COAD project samples and its mean value.

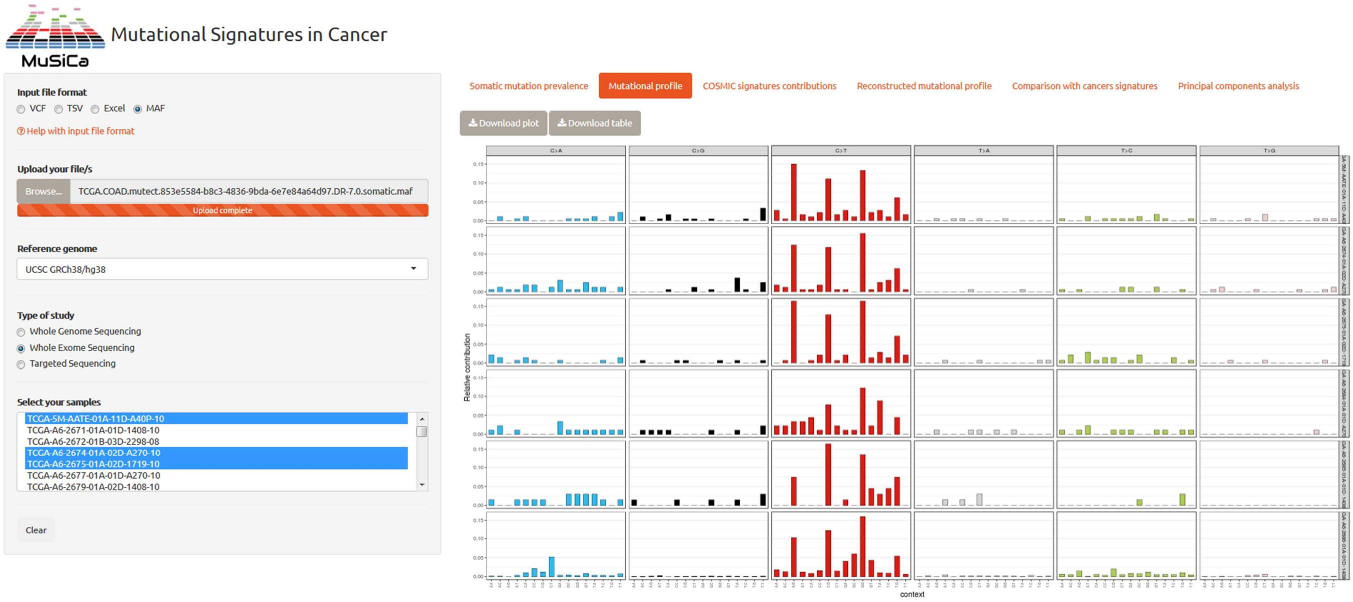

Supplementary Fig. 2. Mutational profile representation in MuSiCa web app. Mutational profile output tab showing a subset of the TCGA-COAD samples.

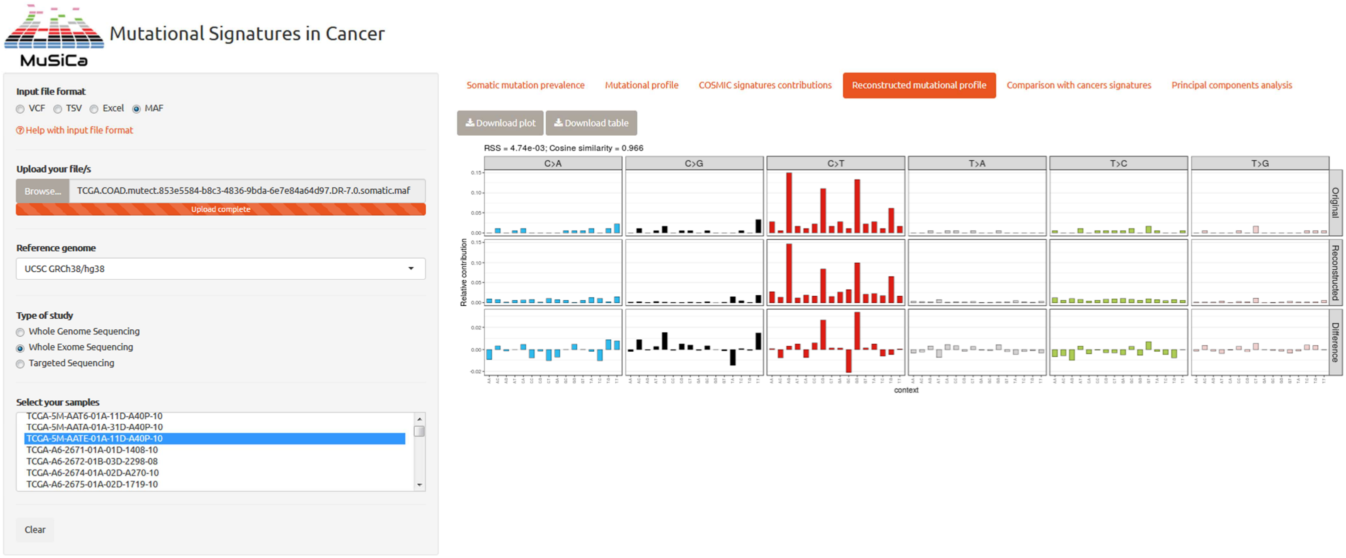

Supplementary Fig. 3. Reconstruction of mutational profile in MuSiCa web app. Reconstructed mutational profile output tab showing a specific sample of the TCGA-COAD project.

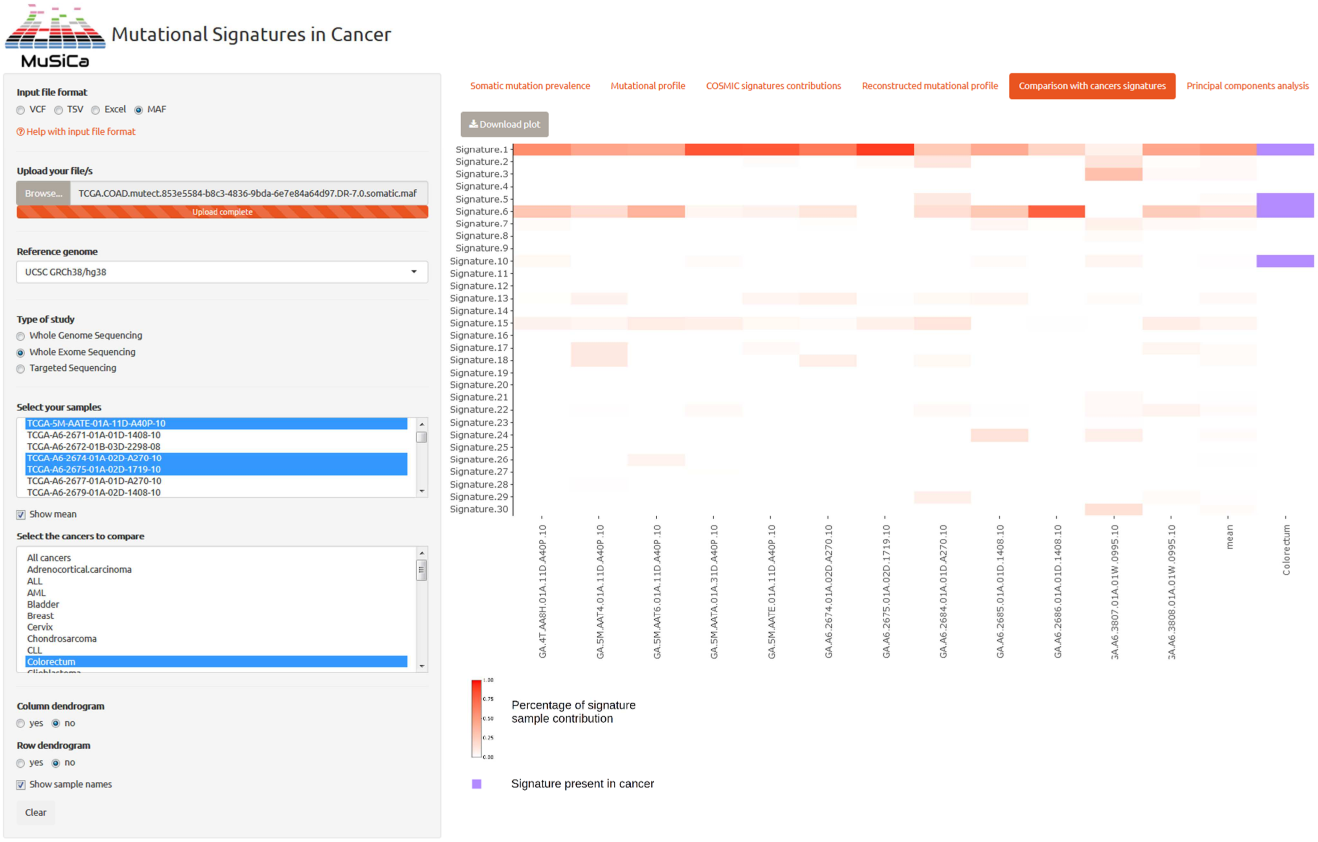

Supplementary Fig. 4. Comparison with cancer signatures in MuSiCa web app. Output tab presenting a comparison of known signatures contributions with mutational signatures reported in different human cancer types in a subset of the TCGA-COAD samples and its mean value.

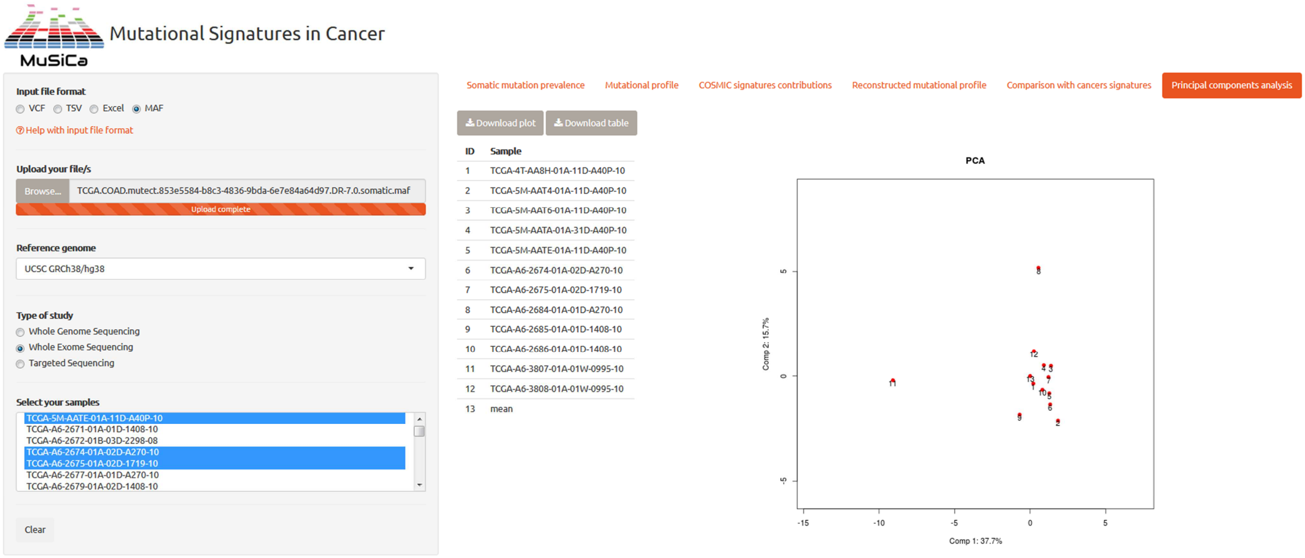

Supplementary Fig. 5. Principal component analysis in MuSiCa web app. Principal component analysis output tab presenting a classification of a specific subset of the TCGA-COAD samples according to the quantification of known signatures contributions.
